# Illness Severity Moderated Association Between Trait Anxiety and Amygdala-Based Functional Connectivity in Generalized Anxiety Disorder

**DOI:** 10.3389/fnbeh.2021.637426

**Published:** 2021-03-18

**Authors:** Yang Du, Hailong Li, Hongqi Xiao, Mei Wang, Wei Zhang, Qiyong Gong, Changjian Qiu, Xiaoqi Huang

**Affiliations:** ^1^Mental Health Center and Psychiatric Laboratory, The State Key Laboratory of Biotherapy, West China Hospital of Sichuan University, Chengdu, China; ^2^Functional and Molecular Imaging Key Laboratory of Sichuan Province, Department of Radiology, Huaxi MR Research Center, West China Hospital, Sichuan University, Chengdu, China; ^3^Psychoradiology Research Unit of the Chinese Academy of Medical Sciences (2018RU011), West China Hospital of Sichuan University, Chengdu, China

**Keywords:** generalized anxiety disorder, amygdala, resting-state functional connectivity, trait anxiety, moderating effect

## Abstract

Trait anxiety is considered a vulnerability factor for the development of generalized anxiety disorder (GAD). The amygdala is related to both trait anxiety and GAD. Thus, we investigated amygdala-based functional connectivity (FC) in drug-naive non-comorbid GAD patients and explored its associations with personality, symptoms, and illness severity. FC analyses using the bilateral amygdala as seeds were performed with resting-state functional MRI data from 38 GAD patients and 20 matched healthy controls (HCs). Clinical characteristics were correlated with FC *Z*-scores from regions showing significant group differences. Furthermore, moderation analyses were used to explore the conditional effect of illness severity measured by the Clinical Global Impression–Severity (CGI-S) scale on the relationship between FC and trait anxiety. Relative to HCs, GAD patients showed hypoconnectivity between the amygdala and the rostral anterior cingulate cortex (rACC), inferior frontal gyrus (IFG), parahippocampal gyrus, and cerebellum and hyperconnectivity between the amygdala and the superior temporal gyrus (STG), insula, and postcentral gyrus. In GAD patients, amygdala–rACC connectivity was negatively associated with symptom severity and trait anxiety, and amygdala–IFG connectivity was positively associated with symptom severity. Moreover, CGI-S scores moderated the negative correlation between trait anxiety and amygdala–rACC FC. We demonstrate that there is extensive amygdala-based network dysfunction in patients with GAD. More importantly, amygdala–rACC connectivity plays a key role in the neural pathology of trait anxiety. Finally, the more severe the illness, the stronger the negative association between trait anxiety and amygdala–rACC FC. Our results emphasize the importance of personalized intervention in GAD.

## Introduction

Trait anxiety, a relatively stable and permanent characteristic of individuals, is characterized by vulnerability to worrying, irritability, and susceptibility to emotionally ambiguous stimuli (Reiss, 1997). High trait anxiety is closely associated with an increased risk of numerous mental disorders, particularly generalized anxiety disorder (GAD) (Gomez and Francis, 2003). With lifetime prevalence rates of 4–7% (Hoge et al., 2012), GAD is a common disorder that causes serious impairment of social function and increases the risk of suicide (De la Vega et al., 2018). Despite the high prevalence and clinical importance of GAD, its neural basis and its association with trait anxiety and clinical profiles remain unclear and deserve to be clarified.

Resting-state functional connectivity (rsFC) holds promise to reflect stimulus-independent correlations of intrinsic activity within the brain (Smitha et al., 2017). The amygdala plays a vital role in the “anxiety circuit” (Tovote et al., 2015), and it is demonstrated that disruptions in functional connectivity (FC) between the amygdala and emotion modulation regions, such as the medial prefrontal cortex (mPFC) and rostral anterior cingulate cortex (rACC), contribute to the neuropathology of GAD (Duval et al., 2015). For example, one study finds that, compared with healthy controls (HCs), GAD patients demonstrate decreased amygdala-based functional connectivity (amyFC) with the dorsal/midcingulate cortex and increased amyFC with the mPFC (Etkin et al., 2009). Previous studies reveal decreased amyFC with the dorsolateral prefrontal cortex (DLPFC) and subgenual anterior cingulate cortex (sgACC) in adolescent GAD patients (Roy et al., 2013; Liu et al., 2015). Reduced connectivity between the amygdala and the rACC (Pace-Schott et al., 2017) is also reported in GAD patients with comorbidities of other anxiety-related disorders. Most previous studies have been carried out with patients with comorbidities or on drug treatment, which may interfere with cerebral function.

Evidence from neuroimaging studies has indicated that trait anxiety is associated with altered amyFC patterns in healthy subjects (He et al., 2016). It is suggested that trait anxiety and GAD may share a common underlying pathology, and anxiety can be conceptualized as a dimensional rather than a categorical construct (Endler and Kocovski, 2001). Trait anxiety impacts the development of anxiety disorders, possibly through concurrent alterations in neural circuitry (Kim et al., 2016). A negative association was observed between amygdala–PFC FC and severity of anxiety in GAD patients during task-based fMRI (Makovac et al., 2016). Another study describes an inverted U-shaped association between anxiety severity and amygdala–PFC FC and suggests that moderate anxiety is associated with maximal amygdala–PFC connectivity in elderly individuals (Wu et al., 2019). Illness severity measured by the Clinical Global Impression–Severity (CGI-S) scale is an overall summary assessment, including symptom severity, the patient's level of distress and other aspects of impairment, and the impact of the illness on functioning (Berk et al., 2008). Based on existing findings, different levels of illness severity may arise from different amyFC in GAD patients. In addition, trait anxiety may be associated with GAD illness severity by bidirectionally affecting each other's manifestation (Gomez and Francis, 2003). Therefore, the illness severity of GAD may have a conditional effect on the association between amyFC and trait anxiety in GAD patients, which should not be overlooked. However, no study has attempted to clarify these interactions and the potential moderating effect between trait anxiety personality and amyFC in GAD patients.

Thus, in the current study, we aimed to explore alterations in amygdala-based rsFC in drug-naive GAD patients without comorbidities. Based on the close relationship between trait anxiety and GAD in pathophysiology and neurobiology, we hypothesized that trait anxiety is related to aberrant amyFC in GAD patients and may be moderated by the severity of the illness.

## Materials and Methods

### Participants

A total of 38 patients with GAD and 20 healthy participants matched for age, sex, and years of education were enrolled in this study. The retrospective study was approved by the ethics committee of the West China Hospital, Sichuan University, and all participants provided written informed consent after they were given a description of this study. Every participant was right-handed and a native Chinese speaker. GAD patients were recruited from the Mental Health Center, West China Hospital, Sichuan University, and diagnoses were based on the Mini International Neuropsychiatric Interview (MINI), Chinese version (Tianmei et al., 2009), by two experienced psychiatrists. The exclusion criteria included (1) age younger than 18 years or older than 65 years; (2) psychiatric comorbidity assessed using the MINI; (3) history of major physical illness, cardiovascular disease, or psychiatric or neurological disorder; (4) substance abuse or dependence; and (5) pregnancy. The Hamilton Anxiety Scale (HAMA) (Maier et al., 1988) and the Generalized Anxiety Disorder 7-item Scale (GAD-7) (Spitzer et al., 2006) were used to measure symptom severity (Ruiz et al., 2011; Thompson, 2015), and the CGI-S was used to evaluate illness severity (Berk et al., 2008). The State-Trait Anxiety Inventory (STAI) (Shek, 1988) was used to assess anxiety-related personality traits.

### Image Acquisition

Resting-state fMRI was performed via a 3-Tesla Siemens MRI system with an 8-channel phase-array head coil. Foam pads were used to reduce head motion and scanner noise. Prior to the scan, the subjects were instructed to keep their eyes closed, relax but not fall asleep, and move as little as possible during scanning. The images were obtained via a gradient-echo echo-planar imaging sequence with the following parameters: repetition time (TR) = 2,000 ms, echo time (TE) = 30 ms, flip angle = 90°, slice thickness = 5 mm with no slice gap, field of view = 240 × 240 mm^2^, 30 axial slices, and 205 volumes in each run.

A high-resolution T1-weighted 3-D spoiled gradient recall (SPGR) sequence was used with the following parameters: TR = 1,900 ms, TE = 2.28 ms, flip angle = 9°, 176 sagittal slices with slice thickness = 1.0 mm, field of view was 240 × 240 mm^2^, and data matrix was 256 × 256.

### Image Preprocessing

In the present study, we applied the Data Processing Assistant for Resting-State fMRI software (DPARSF, http://www.restfmri.net, version 2.1) to perform the image preprocessing. We discarded the first 10 time points to ensure signal stabilization. Slice timing and head motion correction were then conducted. To minimize the effects of head motion on FC, we used the Friston 24-parameter model, which has been suggested to be superior to the 6-parameter model (Friston et al., 1996; Yan et al., 2013). All the subjects were under the threshold of spatial movement in any direction <2 mm or 2°, and the mean framewise displacement was <0.5 mm. Next, the images were normalized to the standard Montreal Neurological Institute (MNI) space, and each voxel was spatially resampled to a voxel size of 3 × 3 × 3 mm^3^. Subsequently, the linear trend of the rs-fMRI data was removed, and band-pass filtering (0.01–0.08 Hz) was conducted to decrease the effect of high-frequency physiological noise and the systematic drift. Furthermore, we regressed out nuisance covariates, including the head motion parameters and the signals from the cerebrospinal fluid and white matter to reduce the effects of non-neuronal BOLD fluctuations.

### Functional Connectivity Analysis

The Resting-State fMRI Data Analysis Toolkit (REST) (http://www.restfmri.net/forum, version 1.8) was used for the calculation of FC. Seed-based resting-state FC of the bilateral amygdala was performed. Bilateral amygdala masks were created using the SPM Anatomy Toolbox (www.fz-juelich.de/inm/inm1/DE/Forschung/_docs/SPMAnatomyTool box/SPMAnatomyToolbox_node.html) (Eickhoff et al., 2005). Pearson's correlation coefficients were computed between the mean time course of each seed and voxel across the whole brain. The voxel-wise Pearson's correlation coefficients were then *z*-scored using Fisher's *Z* transform for subsequent statistical analyses.

### Statistical Analysis

We performed voxel-based comparisons of *Z*-score FC maps of the bilateral amygdala between the two groups using a random-effects two-sample *t*-test in SPM8, which identified brain regions showing significant connectivity differences with each seed in the GAD patients relative to the HCs. Statistical analyses utilized a statistical height threshold of *p* < 0.005 uncorrected at the voxel level and AlphaSim correction (*p* < 0.05) at the cluster level.

### Correlation and Moderation Analysis

Correlations with symptom severity evaluated by HAMA and GAD-7 scores and personality traits measured by STAI scores were examined by extracting FC *Z*-scores from regions showing group differences and correlating these with HAMA, GAD-7, and STAI scores in the GAD group. Moderation analyses were conducted using the PROCESS macro of Hayes (Hayes, 2013) to verify the moderating effect of the interaction between trait anxiety and illness severity shown by CGI-S. A 95% confidence interval (CI) was included in the PROCESS macro for the interaction term, which involved running the analyses with 1,000 bootstrapped samples. The significant effect was indicated by a 95% CI not containing zero. Simple effects examined the associations between trait anxiety and amygdala–rACC FC at low (−1 SD) and high (+1 SD) scores from the mean illness severity.

## Results

### Demographic and Clinical Characteristics

The demographic and clinical characteristics of the two groups are presented in Table 1. There was no group difference in age, sex, or education level between the GAD patients and HCs. For the 38 GAD patients, the HAMA score was 26.82 ± 6.74, GAD-7 score was 12.61 ± 5.13, State Anxiety Inventory (SAI) score was 53.89 ± 11.43, Trait Anxiety Inventory (TAI) score was 54.37 ± 8.53, and CGI-S score was 3.79 ± 0.811. The scores of the clinical rating scales for the GAD patients were significantly different from those in HCs. The estimated duration of GAD symptoms was 52.58 ± 63.121 months.

**Table 1:** Demographic and clinical characteristics.

	**GAD (*n* = 38)**	**HC (*n* = 20)**	***p*-value**
Age (years)	41.11 ± 11.17	39.15 ± 8.24	0.453
Sex (M/F)	10/28	3/17	0.509
Education (years)	11.82 ± 3.88	13.70 ± 3.29	0.070
Duration (months)	52.85 ± 63.12	–	–
HAMA	26.82 ± 6.74	0.05 ± 0.22	<0.001
GAD-7	12.61 ± 5.13	1.30 ± 1.98	<0.001
SAI	53.89 ± 11.43	34.05 ± 7.46	<0.001
TAI	54.37 ± 8.53	35.55 ± 7.37	<0.001
CGI-S	3.79 ± 0.811	–	–

*HAMA, Hamilton Anxiety Scale (14-item); GAD-7, Generalized Anxiety Disorder (7-item); SAI, State Anxiety Inventory; TAI, Trait Anxiety Inventory; CGI-S, Clinical Global Impression-Severity scale. Data are presented as the mean ± SD*.

### Functional Connectivity

**Figure 1:**
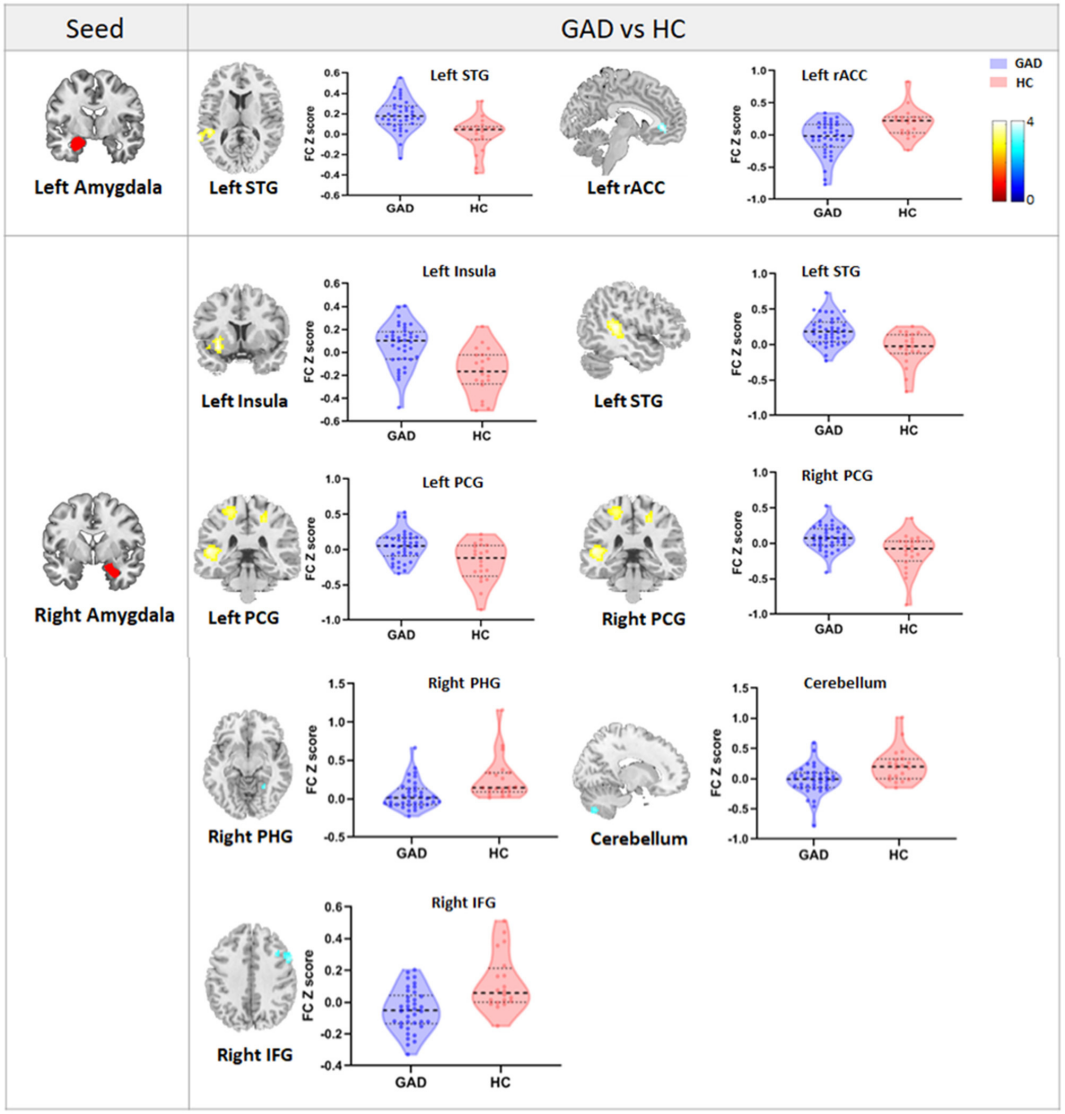
The figure shows the group differences in FC between the bilateral amygdala and the whole brain. Violin plots reflect the mean FC *Z*-scores in each group, and error bars represent standard errors of the means. Warm/cold color indicates increased/decreased FC in GAD patients compared with HCs. STG, superior temporal gyrus; rACC, rostral anterior cingulate cortex; PCG, postcentral gyrus; PHG, parahippocampal gyrus; IFG, inferior frontal gyrus.

Regarding the left amygdala, we found decreased FC (hypoconnectivity) between the left amygdala and the left rACC and increased FC (hyperconnectivity) between the left amygdala and the left superior temporal gyrus (STG) in GAD patients compared with HCs (Table 2, Figure 1). Regarding the right amygdala, increased FC existed between the right amygdala and left insula, left STG, and bilateral postcentral gyrus. Additionally, we found decreased FC between the right amygdala and cerebellum, right parahippocampal gyrus (PHG), and right inferior frontal gyrus (IFG) in GAD patients compared with HCs (Table 2, Figure 1).

**Table 2:** Group comparison of functional connectivity between the bilateral amygdala and whole brain.

	**Cluster location**	**MNI coordinates (x, y, z)**	**Cluster size**	***T*-value**
**SEED: LEFT AMYGDALA**				
GAD > HC				
	Left superior temporal gyrus	−66, −45, 12	151	3.84
GAD < HC				
	Left rostral anterior cingulate cortex	−9, 36, 0	54	−3.54
**SEED: RIGHT AMYGDALA**				
GAD > HC				
	Left insula	−30, 9, −12	248	4.15
	Left superior temporal gyrus	−48, −36, 9	180	4.06
	Left postcentral gyrus	−24, −33, 60	68	3.53
	Right postcentral gyrus	21, −33, 51	52	3.53
GAD < HC				
	Cerebellum	15, −71, −47	110	−3.83
	Right parahippocampal gyrus	27, −54, −9	62	−3.44
	Right interior frontal gyrus	54, 9, 39	75	−3.37

### Correlation and Moderation Analyses

**Figure 2:**
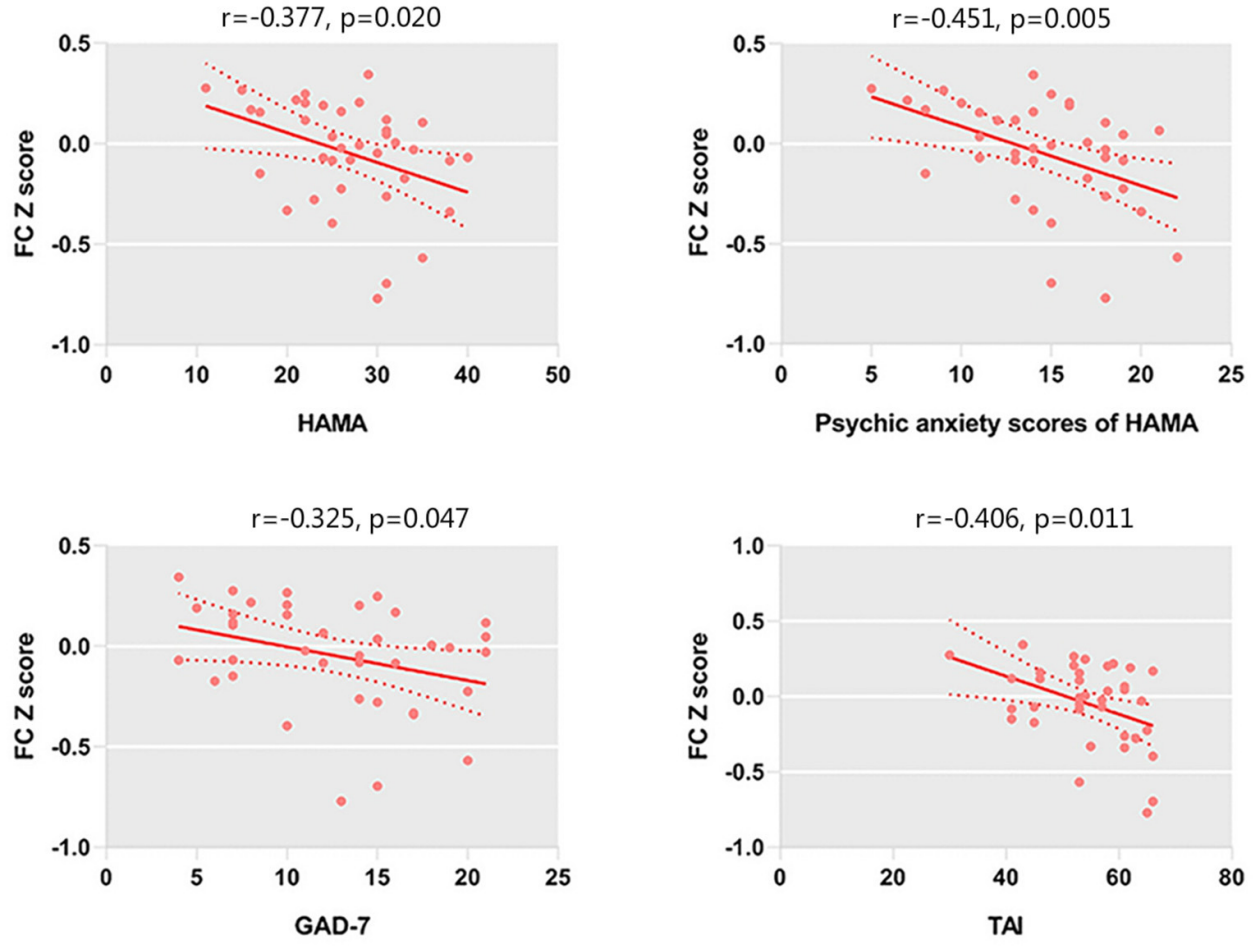
FC between the left amygdala and left rACC is negatively correlated with the HAMA scores, psychic anxiety scores of the HAMA, GAD-7 scores, and TAI scores.

**Figure 3:**
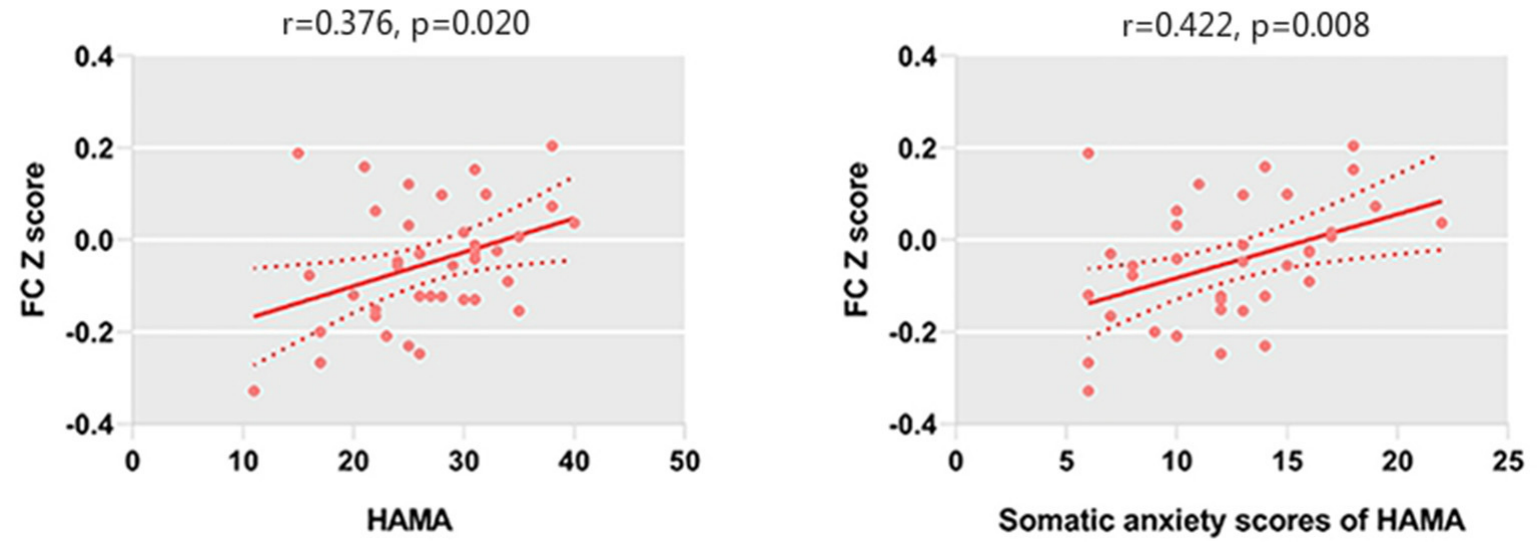
HAMA scores and somatic anxiety scores of the HAMA are positively correlated with the FC between the right amygdala and right IFG.

We only observed a negative correlation between TAI scores and left amygdala–rACC FC (*r* = −0.406, *p* = 0.011) in GAD patients. In addition, left amygdala–rACC FC was negatively associated with HAMA total scores (*r* = −0.377, *p* = 0.020), psychic anxiety scores of the HAMA (*r* = −0.451, *p* = 0.005) and GAD-7 scores (*r* = −0.325, *p* = 0.047) (Figure 2). Furthermore, we found that FC between the right amygdala and the right IFG positively correlated with HAMA total scores (*r* = 0.376, *p* = 0.020) and somatic anxiety scores of the HAMA (*r* = 0.422, *p* = 0.008) in GAD patients (Figure 3).

**Figure 4:**
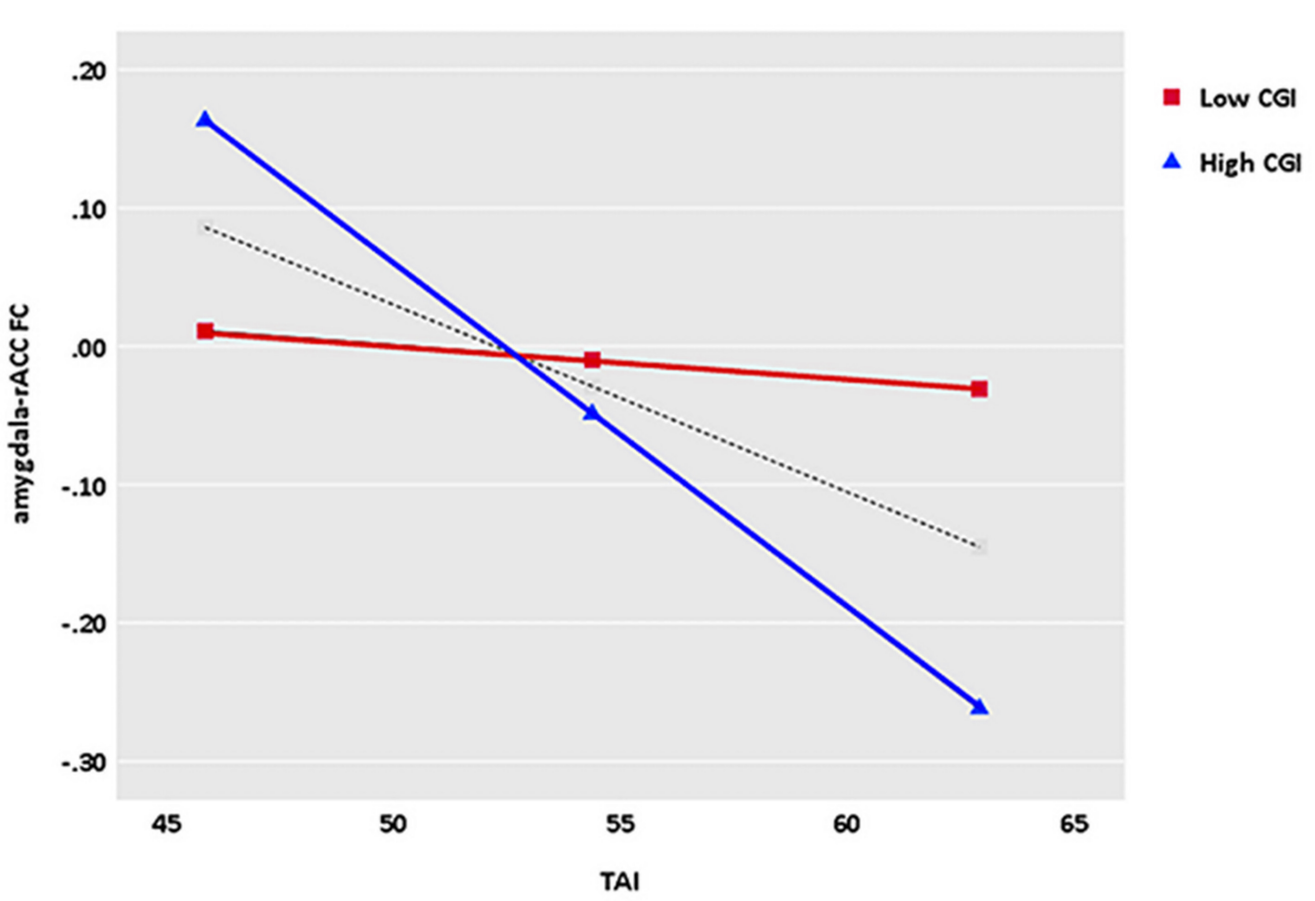
The CGI-S scores moderate the relationship between trait anxiety and amygdala–rACC FC.

Illness severity as measured by the CGI-S moderated the relationship between trait anxiety and left amygdala and rACC FC (bootstrapped 95% CI = −0.026, −0.002). Simple effects depicting the moderating effect of illness severity in relation to trait anxiety and amygdala–rACC FC are presented in Figure 4. The significant interactive effect indicate that the slopes of those simple effects significantly differ (Huberty, 1992), suggesting that the strength of the relation between trait anxiety and amygdala–rACC FC was stronger as illness severity measured by the CGI-S increased.

## Discussion

In the current study, we found that GAD patients had aberrant amyFC with an extensive range of brain regions involved in emotional and cognitive processing. More precisely, hypoconnectivity was found between the left amygdala and the rACC, and hyperconnectivity was observed between the left amygdala and the STG. Additionally, hypoconnectivity was found between the right amygdala and cerebellum, PHG, and IFG, and hyperconnectivity was displayed between the right amygdala and insula, STG, and PCG. In addition, there was a negative association between the left amygdala and rACC connectivity and symptom severity and trait anxiety and a positive association between the right amygdala and IFG connectivity and symptom severity in GAD patients. The severity of illness as reflected by the CGI-S scores moderated the negative correlation between amygdala–rACC FC and trait anxiety, which indicated that the more severe the illness, the stronger the negative association between amygdala–rACC FC and trait anxiety.

We observed decreased left amyFC with the rACC in GAD patients compared with HCs. The rACC has been identified as a distinctive region anatomically and functionally connected with the amygdala, which exerts an inhibiting influence on the amygdala and plays a “top-down” regulatory role in the cognitive control of emotion (Etkin et al., 2011). The failure of engaging the rACC to inhibit amygdala activity and decreased connectivity between the amygdala and the rACC during emotional processing tasks is observed in GAD patients (Etkin et al., 2010; Swartz et al., 2014). Furthermore, a structural magnetic resonance study indicated that reduced structural connectivity between the amygdala and rACC may provide a structural basis for the neuropathological mechanisms of GAD (Tromp et al., 2012). In evidence from a psychotherapy study, GAD patients following mindfulness training showed a significant increase of amygdala–rACC FC from pre- to post-intervention corresponding with symptom improvement, suggesting that effective psychotherapy can change the hypoconnectivity of amygdala–rACC in GAD patients before treatment (Holzel et al., 2013).

Our finding of decreased connectivity between the left amygdala and rACC provides additional information on the role of the rACC in GAD pathology from a network point of view. More importantly, we demonstrate that decreased amygdala–rACC FC is associated with increased trait anxiety in patients with GAD. Previous studies show that healthy individuals presenting higher trait anxiety had weaker amygdala structural and FC with prefrontal regions involved in emotion modulation (Kim et al., 2011; Greening and Mitchell, 2015). To the best of our knowledge, our study is the first to demonstrate a similar association in GAD patients, which may provide evidence for the role of amygdala–rACC connectivity in the boundaries between healthy and pathological anxiety.

In addition, left amygdala–rACC FC was negatively associated with HAMA total scores (*r* = −0.377, *p* =0.020), psychic anxiety scores of the HAMA (*r* = −0.451, *p* = 0.005), and GAD-7 scores (*r* = −0.325, *p* = 0.047). However, the correlation between left amygdala–rACC FC and the somatic anxiety scores of the HAMA showed no significance. Generally, both the GAD-7 and the HAMA are widely used measures that assesses the severity of global anxiety. Indeed, the psychic anxiety scores of the HAMA could be a better indicator of psychological anxiety (Thompson, 2015). Our findings suggested that amygdala–rACC FC is associated with the severity of anxiety, especially psychological anxiety but not somatic anxiety. Taken together, our results reinforce the important role of amygdala–rACC FC in the neural pathology of GAD.

Another important finding in the current study worth noting is the moderate effect of illness severity on the relationship between trait anxiety and left amygdala–rACC FC. We found that the greater the illness severity as measured by CGI-S scores, the stronger the negative correlation between trait anxiety and left amygdala–rACC connectivity, which reflects the sharply increased negative association between trait anxiety and left amygdala–rACC FC as illness severity is enhanced. Conversely, the change amplitude of the negative correlation between trait anxiety and left amygdala–rACC FC is relatively low in mild GAD patients. In general, the negative association between trait anxiety and left amygdala–rACC FC is not static or fixed but is moderated by severity to varying degrees. Furthermore, the findings can be utilized for clinical practice for personalized intervention. Based on our findings and the abovementioned neural network in anxiety, GAD patients with lower trait anxiety and mild illness severity are associated with weaker amygdala–rACC connectivity, which means that brief therapies, such as psychotherapy and moderate physiotherapy, may be suitable for those patients (Garfinkle and Behar, 2012). On the contrary, GAD patients with higher trait anxiety and greater illness severity are related to stronger negative amygdala–rACC connectivity, which means that timely and powerful interventions, such as effective pharmacotherapy, should be taken by psychiatrists (Strawn et al., 2018). It is noteworthy that GAD patients with mild illness severity had a slight decrease in amygdala–rACC connectivity as trait anxiety increased, suggesting that moderate treatment should be considered first in this situation. Therefore, the results revealed in our exploratory analysis may provide novel insights into clinical evaluation and timely intervention for different clinical characteristics of GAD patients. Furthermore, amygdala–rACC FC plays a particularly important role in pathological anxiety, which could be a potential and promising therapeutic “brain target” for GAD.

In addition, GAD patients demonstrate decreased right amyFC with the PHG. The PHG has multiple and direct connections with the amygdala and plays a crucial role in emotion processing and memory encoding (Almeida et al., 2009). The PHG is a key region in the default-mode network (DMN), which is mainly associated with contextualization of safety memories and constrains the generalization of fear (Marstaller et al., 2017). Decreased amyFC with the PHG may cause difficulty in evaluative processing and information integration responsible for generalized worry in GAD patients.

We observed increased bilateral amyFC with the STG in adult GAD patients. Other studies also found increased right amyFC with the STG in adolescent patients with GAD (Roy et al., 2013; Liu et al., 2015). Notably, adult GAD patients showed bilateral amyFC alterations, and adolescent GAD patients merely demonstrated right amyFC changes. Evidence from the lateralization of the amygdala shows that the right amygdala may be more strongly engaged than the left amygdala in a fast analysis of affect-related information (Markowitsch, 1998). Therefore, we hypothesize that the amygdala-based FC network may suffer from more extensive impairment in adult patients. Additionally, the STG contributes to word recognition, language comprehension, and emotional expression perception (Phillips et al., 1998). Activation in the STG is related to emotion regulation in patients with mood and anxiety disorders (Pico-Perez et al., 2017). It is shown that increased spontaneous activity in the STG was found in GAD patients (Xia et al., 2017). Increased rsFC between the amygdala and STG may result in impertinent understandings and exaggerated emotional responses toward external information in GAD patients. The rACC, PHG, and STG are all components of the DMN (Raichle, 2015), which is hypothesized to perform functions, such as emotion regulation, self-referential activities, and self-inspection (Sylvester et al., 2012). Decreased FC between portions of the DMN and the amygdala was found in patients with GAD during emotional conflict adaptation tasks (Etkin et al., 2010). On account of current findings, we can speculate that disrupted amyFC with the DMN plays a major role in the neural mechanisms of GAD.

Patients with GAD also show increased right amyFC with the insula. As the hub in the salience network (SN), the insula receives multifaceted sensory input ranging from interoceptive states to environmental events, exerting multimodal convergence through reciprocal connections with the amygdala and other limbic areas (Menon and Uddin, 2010). Somatic anxiety is a common symptom of GAD, which has always been characterized by a diversity of clinical symptoms involving almost all systems of the body, such as palpitation, anhelation, dysphagia, and dizziness. Increased amyFC with the insula may result in excessive perception and unnecessary responses to subliminal stimulation, constituting a possible cause for the common complaints about multifarious physical discomfort in GAD patients. Previous studies demonstrate increased FC between the right amygdala and the insula in adolescents with GAD (Roy et al., 2013; Hamm et al., 2014; Liu et al., 2015). Elevated activity of both the amygdala and insula in adults with GAD is confirmed by event-related task fMRI (Buff et al., 2016). Our results implicate dysfunction between the amygdala and insula in the resting state in the etiology and maintenance of GAD.

Increased right amyFC with the bilateral postcentral gyrus (PCG) is also found in the current study. The PCG is the hub of the sensorimotor network (SMN) and is responsible for receiving sensory information from distinct brain regions (Li et al., 2019). Increased amyFC with the PCG may cause excessive sensory information input leading to hyperesthesia, which is a common sign in GAD patients. Previous studies show that increased amygdala connectivity with the PCG may indicate a predisposition to depression and anxiety (Pagliaccio et al., 2015).

Our results show decreased right amyFC with IFG in GAD patients. The IFG is widely regarded as a functionally diverse region for emotion regulation and cognitive modulation involved in the central executive network (CEN) (Hampshire et al., 2010). GAD patients are associated with flawed threat assessment and cognitive control abilities. Based on our results, we speculate that decreased FC between the amygdala and the IFG may generate the disintegration of emotional processing with executive function. Previous studies have shown functional alterations in the IFG in patients with GAD (Yin et al., 2017; Li et al., 2018) and clinically anxious individuals (Cha et al., 2016). Our findings support abnormal amyFC with the CEN related to emotional and cognitive dysfunction, which may contribute to the pathophysiology of GAD.

Our results show that decreased right amyFC with the cerebellum is also present in GAD patients. In addition to the important component of the neural underpinnings of movement control, the cerebellum takes part in the executive control network implicated in higher cognitive functions, which has been a concern in recent years (Habas et al., 2009). The consistently decreased amygdala connectivity with the cerebellum and the aforementioned IFG were included in the executive control network that contributes to attentional control and cognitive regulation. These findings indicate the incoordination of emotional and cognitive function in patients with GAD. Further studies employing more refined assessments of cognitive function in combination with the executive control network in GAD patients are needed.

There are some limitations in this study. First, we recruited only GAD patients without comorbidities. Although this helps to exclude confounding factors, it also limits our findings from generalizing to the common GAD population, which bears a high rate of comorbidity. Second, the amygdala is a heterogeneous structure encompassing different subregions, each of which may have distinct connectivity patterns with cortical and subcortical regions (Clarke et al., 2019). It is worthwhile to investigate subregional amyFC in future studies.

## Conclusion

In conclusion, our findings indicated that patients with GAD demonstrate extensive amygdala dysfunction with a range of networks involved in the DMN, SN, CEN, and SMN, which suggests inhomogeneous transformations of neural networks leading to abnormal emotional and cognitive processing in GAD. In addition, the novel finding of the amygdala–rACC network in pathological anxiety has provided further insights into the neural mechanisms of GAD. Our study provides the first neurobiological evidence for the moderating effect of illness severity on the association between trait anxiety and the amygdala network, which has indicated the significance of timely and personalized intervention in GAD.

## Data Availability Statement

The raw data supporting the conclusions of this article will be made available by the authors, without undue reservation.

## Ethics Statement

The studies involving human participants were reviewed and approved by the ethics committee of the West China Hospital, Sichuan University. The patients/participants provided their written informed consent to participate in this study.

## Author Contributions

CJQ, XQH, WZ, and QG designed the study. YD, HL, HQX, and MW acquired the data. YD, HL, and HQX analyzed the data. YD and HL wrote the article. CJQ, XQH, WZ, QG, MW, and HQX reviewed. All authors approved the final version for publication.

## Conflict of Interest

The authors declare that the research was conducted in the absence of any commercial or financial relationships that could be construed as a potential conflict of interest.
